# One-minute stair climbing, 50-foot walk, and timed up-and-go were responsive measures for patients with chronic low back pain undergoing lumbar fusion surgery

**DOI:** 10.1186/s12891-019-2512-5

**Published:** 2019-03-30

**Authors:** Max Jakobsson, Helena Brisby, Annelie Gutke, Mari Lundberg, Rob Smeets

**Affiliations:** 1 Department of Orthopaedics, Institute of Clinical Sciences, Sahlgrenska Academy, University of Gothenburg, House R, Göteborgsvägen 31, S-431 80 Mölndal, Sweden; 2 Department of Orthopaedics, Sahlgrenska University Hospital, Gothenburg, Sweden; 3 Division of Physiotherapy, Department of Health and Rehabilitation, Institute of Neuroscience and Physiology, University of Gothenburg, Box 455, S-405 30 Gothenburg, Sweden; 4 Division of Physiotherapy, Department of Neurobiology, Care Sciences and Society (NVS), Karolinska Institutet, Stockholm, Sweden; 5 Department of Rehabilitation Medicine, Research School of CAPRHI, Maastricht University, CIR Revalidatie, Zwolle/Eindhoven, PO Box 616, 6200 Maastricht, MD The Netherlands

**Keywords:** Health outcome assessment, Performance-based measures, Physical performance tests, Functional tests, Sensitivity to change, Minimal important change, Minimal clinically important difference

## Abstract

**Background:**

Physical capacity tasks are useful tools to assess functioning in patients with low back pain (LBP), but evidence is scarce regarding the responsiveness (ability to detect change over time) and minimal important change (MIC). The aim was to investigate the responsiveness and MIC of 5-min walk, 1-min stair climbing, 50-ft walk, and timed up-and-go in patients with chronic LBP undergoing lumbar fusion surgery.

**Methods:**

In this clinimetric study, 118 patients scheduled for lumbar fusion surgery for motion-elicited chronic LBP with degenerative changes were included. All patients performed the physical capacity tasks 5-min walk, 1-min stair climbing, 50-ft walk, and timed up-and-go 8–12 weeks before and six months after surgery. Responsiveness was evaluated by testing five a priori responsiveness hypotheses. The hypotheses concerned the area under the receiver operating characteristics (ROC) curve and correlations (Spearman’s rho) between the change scores of the physical capacity tasks, the Oswestry Disability Index 2.0 (ODI), and back pain intensity measured with visual analog scale (VAS). At least 80% of the hypotheses would have to be confirmed for adequate responsiveness. Absolute and relative MICs for improvement were determined by the optimal cut-off point of the ROC curve based on the classification of improved and unchanged patients according to construct-specific global perceived effect (GPE) scales.

**Results:**

One-minute stair climbing, 50-ft walk and timed up-and-go displayed adequate responsiveness (≥ 80% of hypotheses confirmed), while 5-min walk did not (40% of hypotheses confirmed). The absolute MICs for improvement were 45.5 m for 5-min walk, 20.0 steps for 1-min stair climbing, − 0.6 s for 50-ft walk, and − 1.3 s for timed up-and-go.

**Conclusions:**

The results of responsiveness for 1-min stair climbing, 50-ft walk, and timed up-and-go implies that these have the ability to detect changes in physical capacity over time in patients with chronic LBP who have undergone lumbar fusion surgery.

## Background

Lumbar degenerative conditions, including lumbar spinal stenosis, disc herniation and degenerative disc disease (DDD), are the most common reasons for elective lumbar spine surgery [1, 2]. Over the past two decades, the number of lumbar fusion operations has constantly increased worldwide [3–6].

The outcome of lumbar fusion surgery is often assessed with back-specific patient-reported outcome measures (PROMs) of disability, e.g. the Oswestry Disability Index (ODI). With these, patients rate their perceived limitations in performing various activities commonly affected by low back pain (LBP), such as walking, sitting and lifting [7, 8]. A benefit of back-specific PROMs is that they require little administration and let the patients convey their own view of their health status [9–11]. However, back-specific PROMs have shown low- to very low-quality evidence for content validity [12], meaning that it is not certain whether the activities in the PROMs are those that matter most to the patients themselves. Previous research and clinical experience also indicate discrepancies between patients’ scores on PROMs and how they actually perform activities when observed by others or as measured by wearable equipment (e.g. accelerometers) [13, 14].

Several authors have recommended the use of physical capacity tasks [13, 15–19], during which the patient performs a standardized activity in the clinic rather than self-reporting his/her ability to perform the activity [17]. An example of a physical capacity task is the timed up-and-go, which measures the time it takes for a person to rise from a chair, walk three meters, turn around, walk back to the chair and sit down [17]. Physical capacity tasks have been designed to measure what patients can do in a standardized environment, rather than what they think they can do, and, as such, they appear to capture important information about a patient’s functioning that PROMs do not [17, 20, 21]. Physical capacity tasks have also been suggested to be less influenced by language skills and education level than PROMs [10, 22, 23].

Outcome measures used in clinical practice and research should have sufficient evidence for reliability, validity, and responsiveness to avoid imprecise or biased results in the assessment of health interventions [24–26]. A recent systematic review showed that the physical capacity tasks 5-min walk, 50-ft walk, 1-min stair climbing, and timed up-and-go demonstrated moderate to strong evidence for reliability and validity [27]. However, the review also identified a lack of evidence concerning responsiveness. Responsiveness is one of the most important properties of an outcome measure since it signifies the ability to detect change over time [24]. It has been recommended that responsiveness is investigated by testing a priori hypotheses on expected associations with other instruments [24, 28]. The responsiveness hypotheses of the current study are presented in Table 1.

**Table 1:** A priori responsiveness hypotheses

1.	The change scores (differences between baseline and 6-month assessments) of a physical capacity task will be able to distinguish between patients with and without meaningful improvement^a^ as classified by a construct-specific GPE scale (area under the ROC curve ≥0.70) [24, 37, 56].^b^
2.	The change scores of a physical capacity task will yield greater misclassifications of improved and unchanged patients on a ROC curve when that classification is based on a generic GPE scale rather than construct-specific GPE scales [24, 37, 56].^b^
3.	The change scores of the four physical capacity tasks will be correlated ≥0.50 to each other in the expected direction [17, 22].^c^
4.	The correlations between change scores of physical capacity tasks and the ODI will be at least 0.10 weaker than the correlations between the change scores among the physical capacity tasks themselves [13, 20].
5.	The correlations between change scores of a physical capacity task and VAS on back pain intensity will be at least 0.10 weaker than the correlations between change scores of the physical capacity task and the ODI [13, 17].

*GPE* Global perceived effect, *ODI* Oswestry Disability Index, *ROC* Receiver operating characteristic, *VAS* Visual analog scale

^a^Improved patients were considered to be those who had scored the response alternatives “much better” or “better” on the construct-specific GPE scales and unchanged patients were those who had scored response alternatives “somewhat better,” “unchanged,” or “somewhat worse”

^b^For timed up-and-go, Hypotheses 1 and 2 were tested separately for the construct-specific GPE scales on walking and chair rise, respectively, since the task includes both of these activities

^c^The expected direction depends on whether a negative or positive change score of a physical capacity task indicates an improvement or deterioration. The correlations between five-minute walk and 1-min stair climbing as well as the correlations between 50-ft walk and timed up-and-go were expected to be positive. The other possible correlations among the four physical capacity tasks were expected to be negative

It is also important to determine whether the change over time of an outcome measure is clinically relevant. The minimal important change (MIC), defined as “the smallest change score that patients perceive as important” [28], has been suggested to be a helpful parameter for this purpose [24, 29]. However, the MICs of physical capacity tasks for patients with chronic LBP have been rarely reported in the literature, not least for patients with chronic LBP who undergo lumbar fusion surgery.

The aim was to investigate the responsiveness and MIC of 5-min walk, 1-min stair climbing, 50-ft walk, and timed up-and-go in patients with chronic LBP undergoing lumbar fusion surgery.

## Methods

This clinimetric study had a prospective design using data from a randomized controlled trial (RCT) [30].

### Eligibility criteria

Eligible patients had motion-provoked chronic LBP with degenerative changes of 1–3 lumbar segments, were aged between 18 and 70 years, and were on the waiting list for lumbar fusion surgery [30]. The patients’ main surgical procedure was lumbar fusion surgery for back pain, but they could have minor radiating symptoms with or without a simultaneous surgery for isthmic spondylolisthesis, foraminal stenosis, or disc herniation. Patients with predominant radiculopathy, a rheumatic or neurological disorder, spinal malignancy, thoracolumbar deformities (e.g. idiopathic scoliosis) were excluded. Patients who had undergone decompression surgery for spinal stenosis or those who had a poor understanding of Swedish were also excluded.

### Procedure

Patients were recruited at one university hospital and two private spine clinics in Sweden [30]. An orthopedic surgeon examined the patients and made a diagnosis, based on radiological and clinical findings. The clinic coordinators informed the physiotherapist responsible for patient recruitment when patients were placed on the waiting list. Patients were then contacted by the physiotherapist who informed them of the study and invited them to participate. Patients who were interested in study participation were scheduled for an appointment with an independent observer at one of the private spine clinics, 8–12 weeks before surgery. The independent observer provided the patients with oral and written information about the study. Patients who agreed to participate signed an informed consent form. The independent observer then instructed the patients to fill out PROMs and perform four physical capacity tasks (described below). The patients were then randomized to participation in either a prehabilitation program or conventional care prior to surgery. The prehabilitation program was based on the principles of person-centered care and had a cognitive behavioral approach [30]. The prehabilitation program comprised four preoperative treatment sessions and one postoperative booster session. In accordance with regional procedure, conventional care comprised a single session with a physical therapist. In this session, the patient received information about the post-operative mobilization routine and was introduced to a core exercise program that was initiated the day after surgery. Both study groups received the same physical therapy treatment in the ward after surgery [30]. In the current study, the patients were studied irrespective of the preoperative intervention assigned to them.

Follow-up assessments of the physical capacity tasks for the RCT occurred at 3, 6, 12, and 24 months after surgery [30], but for the purpose of the present study, only the data from baseline and the 6-month follow-up were used.

### Sociodemographic variables and fear-avoidance variables for descriptive statistics

Data on age, gender, education, height and weight, back pain duration, previous back surgery, work status, and comorbidity were collected with the preoperative questionnaire used in the Swedish National Quality Registry for Spine Surgery (Swespine) [2]. The type of surgical procedure and the number of fusion levels were obtained from the patients’ medical journals. Fear of movement, depressive symptoms, and pain catastrophizing were assessed with the Tampa Scale for Kinesiophobia [31], the Hospital Anxiety and Depression Scale [32], and the Pain Catastrophizing Scale [33], respectively.

### Physical capacity tasks

- *5-min walk:* The patient was asked to walk as fast as possible (without running) for a 5-min period [17]. The circuit was 30 m long and octagonal. The distance covered was recorded in meters.
- *1-min stair climbing:* The patient was asked to climb up and down a flight of stairs for one minute [19]. The staircase was straight with ten steps (16 cm high) and with handrails on both sides which the patient was allowed to use. The handrails were positioned too far apart to be used at the same time. The total number of steps was recorded.
- *50-ft walk:* The patient was instructed to walk as fast as possible (without running) until he/she came back to the starting point [17]. The circuit was 15 m (approximately 50 ft) long and figure-of-eight-shaped. The time needed to complete the test was rounded to the nearest 0.1 s.
- *Timed up-and-go:* The patient was asked to rise up from a chair (seat 45 cm high, without armrests) as fast as possible, walk (without running) 3 m to a marked line on the floor, turn around, and walk back to the chair and sit down [17]. The time needed to complete the test was rounded to the nearest 0.1 s.

Five-minute walk, 50-ft walk, and timed up-and-go have demonstrated moderate to strong evidence for adequate test-retest reliability and construct validity [27]. One-minute stair climbing has demonstrated moderate evidence for adequate test-retest reliability [27].

### Anchors in the responsiveness and MIC analyses

- The Oswestry Disability Index 2.0 (ODI) was used to assess patient-reported disability [34]. The ODI has shown a moderate level of evidence of good reliability and construct validity for patients with chronic LBP [35].
- A 100-mm visual analog scale (VAS) was used to assess the intensity of back pain over the last week. The reliability and validity of VAS in patients with chronic pain are supported by previous research [36].
- At the 6-month follow-up, the patient filled out three 7-point construct-specific global perceived effect (GPE) scales on how he/she perceived his/her walking ability, stair climbing ability and chair rise ability to have changed from the baseline assessment to the 6-month follow-up: “much worse,” “worse,” “somewhat worse,” “unchanged,” “somewhat better,” “better,” and “much better” (eAppendix 1). Similar GPE scales have been shown to have good reliability and validity for patients with chronic LBP [37, 38].
- At the 6-month follow-up, the patient filled out a 5-point generic GPE scale on how he/she perceived his/her back pain to have changed from before surgery: “worse,” “unchanged,” “somewhat better,” “much better,” “pain-free.” The scale has shown good responsiveness for patients with chronic LBP undergoing lumbar fusion surgery [39].

### Statistical analysis

Statistical analyses were performed with IBM SPSS, version 24.0 (IBM Corp., Armonk, USA) and R, version 3.5.1 (R Foundation for Statistical Computing, Vienna, Austria). Descriptive statistics were used to characterize demographics and score distributions of the physical capacity tasks and the anchors. Continuous variables were presented as means with standard deviations in case of normal distribution, or medians with interquartile range otherwise. Categorical variables were presented as frequencies with accompanying percentages.

If a patient had missing data for a physical capacity task, the patient was excluded from all the analyses on that particular task. Patients who did not fill out the ODI, VAS, or any of the GPE scales were excluded from the analyses of the responsiveness hypotheses that included that particular outcome measure. In the case of missing data on the GPE scales, patients were also excluded from MIC analyses.

#### Responsiveness analysis

Responsiveness was investigated with a hypothesis-testing approach as recommended by the Consensus-based Standards for the Selection of Health Measurement Instruments (COSMIN) initiative [24]. Responsiveness in the present study was evaluated by testing the five hypotheses presented in Table 1. According to recommendations, an outcome measure is usually considered to have adequate responsiveness if at least 75% of the hypotheses are confirmed [40]: in this study, with five hypotheses, a criterion of at least 80% confirmed was adopted.

Hypothesis 1 was tested by calculating the area under the receiver operating characteristic (ROC) curve for improved and unchanged patients, as classified by the *construct-specific* GPE scales matched for each particular physical capacity task. The area under the ROC curve can vary from 0.5 to 1 and can be understood as the probability of correctly distinguishing improved patients from unchanged, with 1 indicating perfect ability to distinguish improved from unchanged patients [41]. For hypothesis 1, patients scoring “better” and “much better” on the construct-specific GPE scales (matched for each particular physical capacity task) were classified as improved and those scoring “somewhat worse,” “unchanged,” and “somewhat better” were classified as unchanged. Hypothesis 1 was accepted if the area under the ROC curve was ≥0.70 [40]. For timed up-and-go, hypothesis 1 was tested separately for the construct-specific GPE scales on walking and chair rise, since this task includes both of these activities.

Hypothesis 2 concerned the area under the ROC curve for improved and unchanged patients, as classified by the *generic* GPE scale. Patients scoring “much better” and “pain-free” on this scale were classified as improved, and those scoring “unchanged” and “somewhat better” were classified as unchanged. Hypothesis 2 was accepted if the area under the ROC curve generated by the generic GPE scale was lower than the area under the ROC curve generated by the construct-specific GPE scales. For timed up-and-go, hypothesis 2 was tested separately for the construct-specific GPE scales on walking and chair rise,

Hypotheses 3–5 were investigated with Spearman’s rho [42].

#### MIC analysis

MIC for *deterioration* was not calculated for any physical capacity tasks since few patients reported deterioration on the construct-specific GPE scales (*n* = 2). MIC for *improvement* was determined by the optimal cut-off point of the ROC curve based on the classification of improved and unchanged patients according to the construct-specific GPE scales (same dichotomization as for responsiveness hypothesis 1, described above), matched for each specific physical capacity task. The optimal cut-off point of the ROC curve represents the change score of each physical capacity task that yields the smallest number of misclassifications between improved and unchanged patients [43]. Since MIC can be highly influenced by baseline scores [44, 45], relative values were calculated in addition to absolute values. Relative MICs were calculated based on the ROC curve plotted with the percentage of change from baseline of each physical capacity task, and absolute MICs for improvement were calculated based on the ROC curve plotted with the absolute change from baseline for each physical capacity task. The 95% confidence intervals of the absolute and relative MICs for improvement were generated by taking the 2.5 and 97.5 percentiles of the distribution of 10,000 bootstrap samples [46]. This procedure was performed with the R library pROC [47]. Absolute and relative values for MICs for improvement for timed up-and-go were calculated separately for the construct-specific GPE scales for walking and chair rise.

The adequacy of using the construct-specific GPE scales as anchors for the responsiveness and MIC analyses was determined by calculating the correlation (Spearman’s rho) between the construct-specific GPE scales and the change scores of the physical capacity tasks. Previous research suggests that a correlation of at least 0.30 between an anchor and a change score of a measurement instrument is adequate [48].

### Patient characteristics

**Fig. 1:**
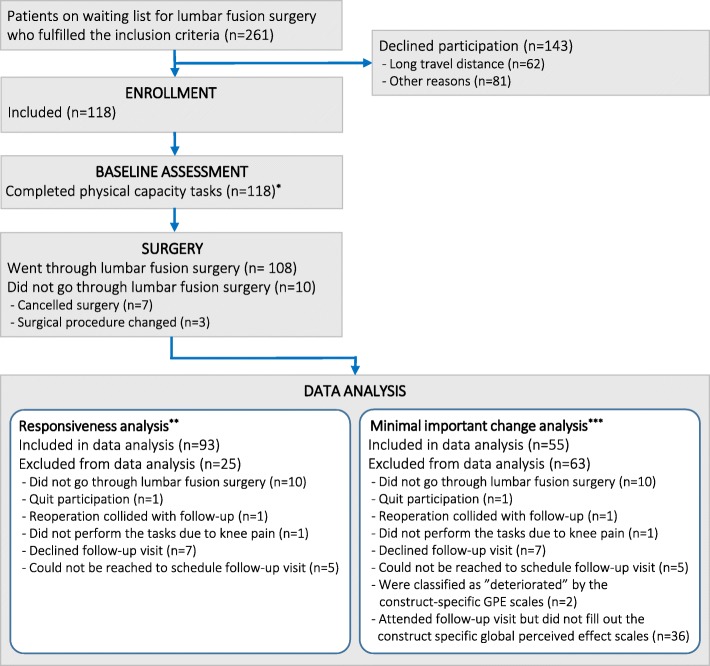
Flowchart of patient inclusion and the number patients included in the responsiveness and minimal important change analyses *One patient did not perform 5-min walk at the baseline assessment. **The number of patients concerns the responsiveness analysis for hypotheses 2–5. In the data analysis for responsiveness hypothesis 1, only 57 patients were included due to missing data on the construct-specific global perceived effect scales. Moreover, since one patient had missing baseline data for 5-min walk, the number of patients in the responsiveness analysis for all hypotheses was one less for this task than for the others. ***For 5-min walk, 54 patients were included in the minimal important change analysis since one patient had missing baseline data for that physical capacity task

Of the 118 included patients, 10 did not go through surgery. Of those undergoing surgery, 15 did not perform physical capacity testing at the 6-month follow-up. The number of patients included in each analysis of responsiveness and MIC for improvement is presented in Fig. 1. Table 2 shows the baseline characteristics of patients who completed the follow-up, and of the drop-outs. Patients in the drop-out group reported significantly higher levels for depressive symptoms, fear of movement, and pain catastrophizing than those completing the follow-up. The frequency of patients who reported disorders that affect walking ability was significantly larger in the drop-out group (four patients) compared with the patients who completed follow-up (two patients). Patients classified as improved by the construct-specific GPE scales had, on average, more favorable changes from baseline of the physical capacity tasks than unchanged patients (Table 3). Average scores for patients classified as deteriorated are not presented in Table 3 due to small sample sizes (*n* = 2).

**Table 2:** Patient characteristics at baseline

Variable	Completed 6-month follow-up of physical capacity tasks (*n* = 93)	Drop-outs (*n* = 25)	Completed 6-month follow-up of physical capacity tasks AND construct-specific GPE scales (*n* = 57)
Age, mean (SD)	46.5 (8.0)	43.0 (9.2)	47.2 (8.4)
Gender, n (%)			
Men	42 (45.2)	13 (52.0)	26 (45.6)
Women	51 (54.8)	12 (48.0)	31 (54.4)
Education level, n (%)			
Elementary school	6 (6.5)	1 (4.0)	5 (8.8)
High school	38 (40.1)	13 (52.0)	22 (38.6)
University or college	36 (38.7)	6 (24.0)	21 (36.8)
Vocational education	12 (12.9)	5 (20.0)	9 (15.8)
Missing information	1 (1.1)	0 (0.0)	0 (0.0)
BMI, mean (SD)	26.2 (3.6)	26.9 (4.0)	26.1 (3.7)
Back pain duration, n (%)			
3–12 months	8 (8.6)	1 (4.0)	3 (5.3)
> 1 year to ≤2 years	17 (18.3)	3 (12.0)	11 (19.3)
> 2 years	66 (71.0)	21 (84.0)	41 (71.9)
Missing information	2 (2.1)	0 (0.0)	1 (1.8)
Previous back surgery, n (%)			
0 occasions	83 (89.2)	24 (96.0)	55 (96.4)
1 occasion	7 (7.5)	0 (0.0)	1 (1.8)
2 occasions	2 (2.2)	1 (4.0)	1 (1.8)
Missing information	1 (1.1)	0 (0.0)	0 (0.0)
Current surgical procedure, n (%)			
Instrumented posterior fusion	88 (94.6)	14 (56.0)	55 (96.5)
Instrumented anterior interbody fusion	1 (1.1)	0 (0.0)	0 (0.0)
Instrumented combined posterior and interbody fusion	4 (4.3)	1 (4.0)	2 (3.5)
Did not go through fusion surgery	0 (0.0)	10 (40.0)	0 (0.0)
Number of fusion levels, n (%)			
One level	55 (59.1)	9 (36.0)	34 (59.7)
Two levels	33 (35.5)	6 (24.0)	21 (36.8)
Three levels	5 (5.4)	0 (0.0)	2 (3.5)
Did not go through fusion surgery	0 (0.0)	10 (40.0)	0 (0.0)
Work status, n (%)			
Working	54 (58.1)	19 (76.0)	33 (57.8)
Part-time sick leave	15 (16.1)	0 (0.0)	9 (15.8)
Fulltime sick leave	17 (18.3)	5 (20.0)	12 (21.1)
Unemployed	4 (4.3)	1 (4.0)	2 (3.5)
Missing information	3 (3.2)	0 (0.0)	1 (1.8)
Comorbidity, n (%)			
Neurological disorder	0 (0.0)	0 (0.0)	0 (0.0)
Heart disease	0 (0.0)	0 (0.0)	0 (0.0)
Other disease that affects walking ability	2 (2.2)	4 (16.0)	2 (3.5)
Other disease that causes pain	4 (4.4)	2 (8.0)	1 (1.8)
5-min walk, mean (SD)	422.3 (82.4)	402.2 (82.4)	419.7 (87.6)
1-min stair climbing, mean (SD)	105.3 (24.8)	99.4 (23.4)	104.9 (26.7)
50-ft walk, mean (SD)	9.2 (2.8)	9.4 (2.6)	9.4 (3.3)
Timed up-and-go, mean (SD)	7.8 (3.0)	8.2 (2.6)	8.2 (3.6)
Disability, ODI, mean (SD)	35.8 (11.5)	40.7 (14.8)	34.6 (12.7)
Back pain intensity, VAS, mean (SD)	60.0 (19.9)	65.1 (17.1)	56.7 (20.5)
Leg pain intensity, VAS, median (IQR)	29.8 (3.4–55.0)	59.5 (7.0–71.4)	29.2 (3.7–59.8)
Depression, HADS, mean (SD)	5.0 (3.5)	6.9 (3.6)	4.6 (3.5)
Kinesiophobia, TSK, mean (SD)	37.3 (8.6)	41.6 (7.1)	37.5 (9.0)
Pain catastrophizing, PCS, mean (SD)	21.8 (7.9)	26.4 (7.9)	22.5 (7.5)

*GPE* Global Perceived Effect Scale, *HADS* Hospital Anxiety and Depression Scale, *IQR* Interquartile range, *ODI* Oswestry Disability Index 2.0, *PCS* Pain Catastrophizing Scale, *TSK* Tampa Scale for Kinesiophobia, *VAS* Visual analog scale

**Table 3:** Baseline, follow-up, and change scores for physical capacity tasks of improved and unchanged patients

Physical capacity task	Construct-specific GPE used for classification of improved/unchanged	GPE categories^a^	Baseline T1 Mean (SD)	Follow-up T2 Mean (SD)	Change score T2-T1 Mean (SD)
5-min walk (m)	GPE_walking_	Improved (*n* = 32)	403.2 (94.2)	484.9 (66.7)	81.7 (75.1)
		Unchanged (*n* = 22)	445.5 (73.3)	474.7 (93.0)	29.2 (59.9)
1-min stair climbing (steps)	GPE_stair climibing_	Improved (*n* = 33)	95.3 (26.9)	125.0 (24.3)	29.7 (23.6)
		Unchanged (n = 22)	114.1 (20.7)	127.1 (22.3)	13.0 (19.4)
50-ft walk (s)^a^	GPE_walking_	Improved (n = 33)	9.9 (3.8)	7.7 (1.5)	−2.2 (3.3)
		Unchanged (n = 22)	8.6 (2.4)	8.3 (2.4)	−0.3 (1.5)
Timed up-and-go (s)^a,b^	GPE_walking_	Improved (n = 33)	9.0	5.8	−3.2 (4.1)
		Unchanged (n = 22)	6.9 (1.6)	6.4 (1.9)	−0.5 (1.6)
	GPE_chair rise_	Improved (*n* = 31)	9.1 (4.4)	5.7 (1.2)	−3.4 (4.0)
		Unchanged (*n* = 28)	7.1 (1.6)	6.4 (1.9)	−0.7 (1.6)

*GPE* Global Perceived Effect scale, *SD* Standard deviation, *T1* Baseline assessment, *T2* 6-month follow-up assessment

^a^A negative score indicates improvement. ^b^The results for timed up-and-go are presented for the classifications of the construct-specific GPE scales for both walking and chair rise since this task includes both these activities.

## Results

### Responsiveness

Hypothesis 1 was confirmed for 1-min walk, 50-ft walk, and timed up-and-go as the areas under the ROC curves generated with the construct-specific GPE scales were ≥ 0.70 for these tasks (Table 4). Hypothesis 2 was confirmed for 1-min walk, 50-ft walk, and timed up-and-go as they had larger areas under the ROC curves generated by the construct-specific GPE scales than those generated by the generic GPE scales (Table 4). In contrast, Hypotheses 1 and 2 were rejected for the 5-min walk. Hypothesis 3 was confirmed for all physical capacity tasks as the correlations among the tasks themselves were ≥ 0.50 (Table 5). Hypothesis 4 was confirmed for all physical capacity tasks as the correlations between the tasks and the ODI were consistently lower than the correlations among the tasks themselves. Hypothesis 5 was rejected for all tasks except for timed up-and-go.

**Table 4:** Area under the receiver operating characteristics curve and minimal important change for the physical capacity tasks

Physical capacity tasks	5-min walk	1-min stair climbing	50-ft walk	Timed up-and-go _GPEwalking_	Timed up-and-go _GPEchair rise_
AUC_Construct-specific GPE_ (95% CI)	0.68 (0.54 to 0.82)	0.72 (0.59 to 0.85)	0.80 (0.67 to 0.93)	0.74 (0.61 to 0.86)	0.79 (0.67 to 0.91)
AUC_Generic GPE_ (95% CI)	0.70 (0.58 to 0.82)	0.70 (0.59 to 0.81)	0.76 (0.66 to 0.87)	0.72 (0.67 to 0.91)	0.72 (0.62 to 0.83)
Correlation between change score and construct-specific GPE	0.39	0.50	−0.57	−0.48	− 0.53
MIC_absolute_ (95% CI)	45.5 m (8.5 to 62.0)	20.0 steps (10.5 to 48.0)	−0.6 s (− 0.7 to − 0.2)	−1.3 s (−2.4 to − 0.5)	−1.3 s (− 2.4 to − 0.3)
Sensitivity for MIC_absolute_	0.63	0.63	0.73	0.67	0.73
Specificity for MIC_absolute_	0.75	0.76	0.83	0.71	0.79
Average absolute change from baseline of the physical capacity tasks, mean (SD)	62.5 m (69.5)	22 steps (22.4)	−1.3 s (2.5)	−2.0 s (2.9)	−2.0 s (2.9)
MIC_relative_ (95% CI)	9.0% (4.5 to 11.8)	12.5% (7.2 to 48.4)	−6.1% (−7.1 to −3.4)	−17.3% (−29.4 to − 10.2)	− 17.6% (− 20.7 to − 10.2)
Sensitivity for MIC_relative_	0.69	0.70	0.76	0.73	0.79
Specificity for MIC_relative_	0.75	0.72	0.88	0.71	0.79

*AUC* Area under the receiver operating characteristics curve, *CI* Confidence interval, *GPE* Global Perceived Effect scale, *MIC* Minimal important change

**Table 5:** Correlations between change scores of physical capacity tasks, Oswestry disability index, and visual analog scale on back pain intensity

	5-min walk	1-min stair-climbing	50-ft walk	Timed up-and-go	ODI	VAS
5-min walk	1.000	0.815^a^	− 0.755^a^	− 0.586^a^	− 0.422^a^	− 0.342^a^
1-min stair climbing		1.000	−0.755^a^	− 0.670^a^	− 0.396^a^	− 0.342^a^
50-ft walk			1.000	0.665^a^	0.467^a^	0.368^a^
Timed up-and-go				1.000	0.413^a^	0.286^a^
ODI					1.000	0.727^a^
VAS						1.000

*ODI* Oswestry Disability Index 2.0, *VAS* Visual Analog Scale on back pain intensity

^a^*p* < 0.01

In summary, one-minute stair climbing, 50-ft walk, and timed up-and-go displayed adequate responsiveness (80% of the hypotheses confirmed for 1-min stair climbing, 50-ft walk, and 100% for timed up-and-go), while 5-min walk did not (only 40% of the hypotheses confirmed) (Table 6).

**Table 6:** Results of Hypothesis-Testing for Responsiveness

Physical capacity tasks	Construct-specific GPE used in Hypotheses 1 & 2	Responsiveness hypotheses confirmed					Percentage of hypotheses confirmed
		1	2	3	4	5	
5-min walk	GPE_walking_	–	–	+	+	–	2 of 5 = 40%
1-min stair climbing	GPE_stair climbing_	+	+	+	+	–	4 of 5 = 80%
50-ft walk	GPE_walking_	+	+	+	+	–	4 of 5 = 80%
Timed up-and-go	GPE_walking_	+	+	+	+	+	5 of 5 = 100%
	GPE_chair rise_	+	+				

*GPE* Global Perceived Effect scale

+, confirmed hypothesis; −, rejected hypothesis

### Minimal important change

Of the 57 patients who completed the construct-specific GPE scales, two reported deterioration on the scales and were excluded from the MIC analyses. Absolute MICs for improvement were 45.5 m for 5-min walk, 20 steps for 1-min stair climbing, − 0.6 s for 50-ft walk, and − 1.3 s for timed up-and-go (Table 4). The sensitivity and specificity of the absolute and relative MICs for improvement are presented in Table 4. As reference values to the MICs for improvement, Table 4 gives the mean change scores of the physical capacity tasks, indicating the change of the “average” patient.

### Adequacy of using the construct-specific GPE scales in the responsiveness and MIC analyses

The correlation between the construct-specific GPE scales and the change scores of the physical capacity tasks were all above the recommended threshold value of 0.30 [48], which supports the adequacy of using the scales in the responsiveness and MIC analyses (Table 4).

## Discussion

The present study was one of the first to assess responsiveness and MIC of physical capacity tasks for patients with chronic LBP undergoing lumbar fusion surgery. One-minute stair climbing, 50-ft walk, and timed up-and-go displayed adequate responsiveness with ≥80% of the responsiveness hypotheses being confirmed, while five-minute walk displayed inadequate responsiveness. The positive results of responsiveness for 1-min stair climbing, 50-ft walk, and timed up-and-go suggests that these physical capacity tasks have the ability to detect changes in physical capacity over time in patients who undergo lumbar fusion surgery. The absolute MICs for improvement for 5-min walk, 1-min stair climbing, 50-ft walk, and timed up-and-go were 45.5 m, 20.0 steps, − 0.6 s, and − 1.3 s, respectively.

In line with our results, Gautschi et al. found adequate responsiveness for timed-up-and-go [49]. Gautschi et al. investigated the responsiveness of timed up-and-go for a mixed study sample of patients with lumbar spinal stenosis, lumbar disc herniation, and chronic LBP due to DDD undergoing various types of lumbar spine operations. In concordance with our findings, Andersson et al. found that one-minute stair climbing had adequate responsiveness [50]. Furthermore, the authors of that study found that five-minute walk had inadequate responsiveness, also in line with our results. Andersson et al. reasoned that the finding might be a result of the possibility that the task was not challenging enough for patients with chronic LBP. Patients might therefore only show small improvements in this task after an intervention, which could limit the task’s responsiveness.

In contrast to our results, Andersson et al. [50] and Strand et al. [51] found that 50-ft walk had inadequate responsiveness. The differences in results might be because of dissimilarities in patient characteristics. Andersson et al. [50] and Strand et al. [51] included patients with non-specific chronic LBP who underwent non-surgical interventions. Patients with chronic LBP undergoing lumbar fusion surgery in the current study had motion-elicited back pain, so that they can have difficulties with quick movements of the spine. As such, 50-ft walk could be challenging for these patients, the task requiring them to make a quick turn after having walked 25 ft. In contrast, the patients in the two previous responsiveness studies [50, 51] may have found the task less challenging. Second, Andersson et al. did not use a hypothesis testing approach to evaluate responsiveness [50], which could also explain why their results differed from ours.

The MICs for improvement in the current study might be used by researchers and clinicians as reference values when interpreting patients’ postoperative change scores [24, 43]. In research, the MICs for improvement could, for example, be used to evaluate the proportion of “responders” to treatment, where patients with change scores larger than the MIC values are classified as responders [52]. It is, however, important to acknowledge that the MIC is a group-based statistic and that the value for MIC might not always reflect an individual patient’s view of the change [53]. Thus, when comparing an individual patient’s change score with the current study’s MICs for improvement in clinical practice, it is essential to interpret the change score in relation to the patient’s reported experience and not just the MIC. Comparing individual change scores with the MICs might, for instance, serve as a reference for what the “average” patient finds important and could possibly aid the shared decision-making process in the patient’s postoperative rehabilitation. However, the 95% confidence intervals of the MICs were wide, and they should therefore be viewed with some caution.

In order to detect changes as small as the MIC, it is important that the MIC is larger than the smallest detectable change (SDC), defined as “the smallest change that can be detected by the measurement instrument, beyond measurement error” [24]. The MIC of 1-min stair climbing in the present study is larger than the SDC (derived from the limits of agreement) in Smeets et al. [19], which suggests that when a patient scores change equal to or greater than the MIC this is indeed an important change and unlikely to be due to measurement error. In contrast, the MICs for improvement of 50-ft walk and timed up-and-go in the present study are below the smallest detectable change given in previous studies [17, 19], meaning that observed changes could be due to measurement error and not reflect important and real changes. As the SDCs have only been assessed in patients with chronic LBP who undergo conservative treatment [17, 19], future studies should investigate the SDC specifically for patients with chronic LBP who undergo lumbar fusion surgery.

A strength of the present study is that it is one of the first to investigate the responsiveness of physical capacity tasks by testing a priori hypotheses. Using a hypothesis testing approach in the assessment of responsiveness has been recommended by experts in clinimetrics since it minimizes bias in the interpretation of the results [24, 28]. Another strength of the study is that we used an anchor-based method (the optimal cut-off point of the ROC curve) to determine MICs. Anchor-based methods have been recommended over so-called distribution-based methods, such as the standardized response mean or other effect size parameters [24]. Moreover, we used construct-specific GPE scales rather than generic GPE scales in the anchor-based method since previous research implies that construct-specific GPE scales generate better approximations of MICs than do generic ones [37]. However, there is no consensus on the optimal method for determining MIC. For instance, the so-called predictive modeling approach has been shown to be a good alternative to the optimal cut-off point method [54]. Research also suggests that MIC estimates may be biased when the proportion of improved patients is higher than 50% (in our study, 60% of the patients were improved) [55]. Consequently, future studies using other methods for determining MIC and also adjusting for the proportion of improved patients might provide better estimates than in the current study.

A limitation of this study is that a large proportion (36 patients) of those whom attended the follow-up visits did not fill out the construct-specific GPE scales. The reason for this is that MIC was first planned to be investigated with a generic GPE scale [39] instead of the construct-specific ones. However, during the course of this study, other studies showed that construct-specific GPE scales seemed to be more suitable for determining MIC [37, 56], and we therefore decided to use this type of scales instead. A natural consequence of this decision is that the results for Hypothesis 1 and the MICs for improvement had less statistical power than the other analyses, which is reflected in the wide confidence intervals of these MICs.

Another limitation could be potential selection bias since the patients were a part of an RCT. Patients with higher preoperative levels of disability and pain intensity may have declined study participation as the RCT required patients to travel to one of the spine clinics to see a physical therapist before surgery [30]. This could be the reason why the study sample reported a slightly lower disability level and back pain intensity compared with patients in Swespine undergoing surgery for chronic LBP due to DDD [2]. However, our study sample had similar characteristics as patients in Swespine in terms of age, duration of symptoms and proportion of men and women. It is therefore reasonable to assume that our findings are generalizable to most patients undergoing lumbar fusion surgery for chronic LBP, but possibly not for those with the highest preoperative levels of disability and pain intensity.

## Conclusions

The results of responsiveness imply that 1-min stair climbing, 50-ft walk, and timed up-and-go they have the ability to detect changes in physical capacity over time in patients with chronic LBP who have undergone lumbar fusion surgery. In contrast, the 5-min walk showed inadequate responsiveness for this patient group.

## Abbreviations

COSMIN: Consensus-based Standards for the Selection of Health Measurement Instruments
DDD: Degenerative disc disease
GPE: Global perceived effect
LBP: Low back pain
MIC: Minimal important change
ODI: Oswestry disability index
PROMs: Patient-reported outcome measures
RMDQ: Roland-Morris disability questionnaire
ROC: Receiver operating characteristic
Swespine: The Swedish National Quality Registry for Spine Surgery
VAS: Visual analog scale
